# Genomic Analyses of Pediatric Acute Lymphoblastic Leukemia Ph+ and Ph-Like—Recent Progress in Treatment

**DOI:** 10.3390/ijms22126411

**Published:** 2021-06-15

**Authors:** Agnieszka Kaczmarska, Patrycja Śliwa, Joanna Zawitkowska, Monika Lejman

**Affiliations:** 1 Student Scientific Society, Laboratory of Genetic Diagnostics, Medical University of Lublin, A. Gębali 6, 20-093 Lublin, Poland; agnieszkakacz70@gmail.com (A.K.); sliwa.pat@gmail.com (P.Ś.); 2 Department of Pediatric Hematology, Oncology and Transplantology, Medical University of Lublin, A. Gębali 6, 20-093 Lublin, Poland; jzawitkowska1971@gmail.com; 3 Laboratory of Genetic Diagnostics, II Chair of Paediatrics, Medical University of Lublin, A. Gębali 6, 20-093 Lublin, Poland

**Keywords:** acute lymphoblastic leukemia, Philadelphia chromosome, *IKZF1*, ALL Ph+

## Abstract

Pediatric acute lymphoblastic leukemia (ALL) with t(9;22)(q34;q11.2) is a very rare malignancy in children. Approximately 3–5% of pediatric ALL patients present with the Philadelphia chromosome. Previously, children with Ph+ had a poor prognosis, and were considered for allogeneic stem cell transplantation (allo-HSCT) in their first remission (CR1). Over the last few years, the treatment of childhood ALL has significantly improved due to standardized research protocols. Hematopoietic stem cell transplantation (HSCT) has been the gold standard therapy in ALL Ph+ patients, but recently first-generation tyrosine kinase inhibitor (TKI)-imatinib became a major milestone in increasing overall survival. Genomic analyses give the opportunity for the investigation of new fusions or mutations, which can be used to establish effective targeted therapies. Alterations of the *IKZF1* gene are present in a large proportion of pediatric and adult ALL Ph+ cases. *IKZF1* deletions are present in ~15% of patients without BCR-ABL1 rearrangements. In BCR-ABL1-negative cases, *IKZF1* deletions have been shown to have an independent prognostic impact, carrying a three-fold increased risk of treatment failure. The prognostic significance of *IKZF1* gene aberrations in pediatric ALL Ph+ is still under investigation. More research should focus on targeted therapies and immunotherapy, which is not associated with serious toxicity in the same way as classic chemotherapy, and on the improvement of patient outcomes. In this review, we provide a molecular analysis of childhood ALL with t(9;22)(q34;q11.2), including the Ph-like subtype, and of treatment strategies.

## 1. Introduction

Acute lymphoblastic leukemia (ALL) is the most common malignancy diagnosed in children according to American Cancer Society data, accounting for 26% of all cancers in children aged 1–14 and 8% in teenagers aged 15–19 in the USA [1]. Over the last few years, the treatment of childhood ALL has improved significantly due to standardized research protocols. The cure rate has increased dramatically and is now about 90% in many developed countries. Identification of chromosomal abnormalities and translocations is important to establish treatment protocols and predict the clinical outcome [2].

The Philadelphia chromosome (Ph), arising from a reciprocal translocation between chromosomes 9 and 22 t(9;22)(q34;q11.2), is present in 3% to 5% of children and 25% of adults with ALL [3]. The normal *BCR* gene encodes a 160-kD phosphoprotein associated with serine/threonine kinase activity. *ABL1* is a proto-oncogene that encodes a cytoplasmatic and nuclear protein tyrosine kinase involved in a variety of cellular processes, including cell adhesion, cell division, cell differentiation, and the stress response [4]. *BCR/ABL1* has a cytoplasmic localization and may have a carcinogenetic role, in contrast with *ABL1*, which has a mostly nuclear localization. The hybrid protein has an increased protein tyrosine kinase (TK) activity compared to *ABL1*. These activated kinases disturb downstream signaling pathways, causing enhanced proliferation, differentiation arrest, and resistance to cell apoptosis. The crucial event lies on der(22); the *5′ BCR/3′ ABL* hybrid gene is pathogenic, while *ABL/BCR* may or may not be expressed. Expression of the fusion gene in ALL results in two types of chimeric mRNAs, dependent upon the position of the breakpoint within the breakpoint cluster region of the *BCR* gene. In a third of patients with ALL and the majority of those with CML, the breakpoint occurs within a 5.8 kb region spanning *BCR* exons 12–16 (exons b1–b5), known as the major bcr (M-bcr). The translocation involving this breakpoint results in the production of a p210 BCR–ABL protein. In the remaining two thirds of ALL patients and rarely in CML, the breakpoint arises further upstream, between exons e2′ and e2, known as the minor bcr (m-bcr). The translocation involving this breakpoint produces a p190 protein. The p190 group (typical for patients with ALL Ph+) tends to show an early response and complete remission at the end of the induction phase, which indicates that patients carrying a p190 transcript have a more favorable prognosis than the p210 group [5].

Historically, the outcome for children with ALL Ph+ was an exceptionally poor prognosis, and all children were classified into the high-risk group. According to the EsPhALL2010 protocol, the overall survival after treatment was 71.8% and event-free survival (EFS) was 57.0% at the five years follow-up. Although current treatment strategies are becoming increasingly successful, the most frequent cause of treatment failure is relapse. Cumulative relapse risk is 9.3–20.6%, and the outcome prognosis after a relapse is poor [6,7,8]. Several studies have clearly demonstrated that minimal residual disease (MRD) also has a crucial predictive role in ALL Ph+ [9,10,11,12]. Moreover, not only the presence of *BCR/ABL1* fusion, but also the age at diagnosis and leukocyte count are adverse prognostic factors associated with a high risk of therapeutic failure [13]. Unfortunately, due to the small number of pediatric ALL Ph+ cases, randomized trials focusing on treatment are still lacking. Genomic analyses give opportunities for the detection of new mutations, which can be used to establish effective targeted therapies [14]. The use of high-throughput analytical techniques has allowed the description of novel high-risk ALL subtypes. One such novel subtype, Ph-like acute lymphoblastic leukemia (Ph-like ALL), is characterized by a spectrum of underlying genetic alterations that activate kinase or cytokine receptor signaling, while lacking the *BCR-ABL1* fusion gene. ALL Ph+ and Ph-like show a high incidence of *IKZF1* alterations. Approximately two-thirds of pediatric B-ALL Ph+ cases have an *IKZF1* deletion, but *IKZF1* point mutations have been identified in up to 10% of *IKZF1* deletion-negative B-ALL Ph+ cases. The presence of *IKZF1* deletions has been associated with an older age at diagnosis, higher presenting white blood cell counts, and higher levels of MRD after induction and consolidation [15]. In this review, we provide the genomic landscape of childhood ALL with t(9;22)(q34;q11.2) and present potential strategies for treatment, and also include consideration of the Ph-like subtype.

## 2. Treatment of Pediatric ALL Ph+

Phosphorylation is the main function of protein tyrosine kinases (PTKs), which are normally absent in the ABL protein. However, when *BCR-ABL1* fusion occurs, PTK functions out of control, damaging the signaling pathways and potentially leading to leukemogenesis. The idea of using a special inhibitor for an exact gene mutation seems to be the most logical option. Imatinib mesylate binds the inactive moiety of BCR-ABL kinase, which completely blocks its ATP binding site, leading to the inhibition of tyrosine phosphorylation of proteins that are involved in the intracellular signal transduction that *BCR-ABL* mediates. This prevents a conformational switch to the active oncoprotein. Using tyrosine kinase inhibitors (TKIs), especially imatinib, increases the chance of an improved outcome in Ph-positive ALL patients. Studies conducted by the Children Oncology Group (COG) and the European EsPhALL consortium have provided evidence that pediatric ALL Ph+ patients can be effectively treated with a combination of chemotherapy and tyrosine kinase inhibitors (TKIs), without HSCT, in the first remission (CR1). To date, three trials have been conducted: EsPhALL2004, EsphALL2010, and EsphALL2017 [16,17]. The first study focused on using post-induction imatinib and chemotherapy in accordance with the Berlin–Frankfurt–Münster protocol. In EsPhALL2004, after induction, patients were classified into two groups: the good risk and poor risk groups. Patients who were classified into the good risk group were randomly allocated imatinib (300 mg/m^2^ per day) plus chemotherapy or chemotherapy alone, whereas those who were categorized into the poor risk group received post-induction imatinib plus chemotherapy. The authors suggested that imatinib can improve patient outcomes in the poor risk group, with minimal residual disease. Any adverse events were mainly related to myelosuppression, rather than to the use of imatinib per se. The effectiveness of HSCT and imatinib treatment remains unclear, however, because the majority of the study group (77%) had HSCT. The second trial, EsPhALL2010, investigated whether continuous administration of imatinib during chemotherapy could give better results compared to EsPhALL2004. In view of ALL Ph+ heterogeneity, patients were divided into two groups: the good risk group, which received HSCT, and the poor risk group, which received only chemotherapy treatment because of the lack of a genotype-matched donor. The major difference to the previous study was that in EsPhALL2010 all patients were continuously given imatinib, along with chemotherapy, from Day 15 of induction to the end of the treatment. EsPhALL2010 also produced opposing results concerning toxicity compared to the EsPhALL 2004 study. There were 14 reported serious events, as well as serious adverse events, which occurred in approximately 52% of all patients. This leads to the conclusion that earlier and longer exposure to imatinib in the course of chemotherapy might increase the toxicity. In the EsPhaLL2010 study, which consisted of administrating imatinib during induction, the results of the first complete remission were 97% compared to 78% in EsPhALL2004, where imatinib was administered after induction. The role of imatinib after HSCT has not been established yet. There were significant differences in exposure to imatinib between the trials. In the EsPhALL2004 study it was 3 or 5 months, depending on whether the patients had transplanted HSCT or not, while in EsPhALL2010, non-transplanted patients were treated for 24 months. The comparison of treatment results without the use of TKIs and the EsPhALL 2004 and 2010 protocols is presented in Table 1. The latest trial, EsPhALL2017, has attempted to balance treatment-related toxicity from intensive chemotherapy, without affecting the high rate of favorable outcomes. From Day 15 of induction, all children with ALL Ph+ received imatinib, and HSCT was performed. Due to MRD patients having been divided into a high-risk group and standard-risk group, MRD was the only factor used in this trial to risk-stratify patients with ALL Ph+. Current clinical trials suggest that chemotherapy plus imatinib could improve the treatment results for ALL Ph+ patients. The EsPhALL2017 study is ongoing, and hopefully its results will represent a breakthrough in ALL Ph+ treatment [18]. Therapeutic failure in ALL Ph+ could be the result of resistance to TKI-based therapies, caused by point mutations in the BCR-ABL1 kinase domain. The generally used Sanger sequencing (SS) for diagnostic BCR-ABL1 kinase domain mutations has some limitations—it has poor sensitivity and is unable to identify compound mutations. Soverini et al. reported the first attempt at defining indications for the use of Next-Generation Sequencing (NGS) for BCR-ABL1 kinase domain mutation screening in ALL Ph+. The authors suggested that NGS results in hopeless cases (relapsed or resistant to treatment ALL), which may have an impact on therapeutic decision-making [19]. However, some studies have indicated that the second-generation tyrosine kinase inhibitors, including dasatinib and nilotinib, should also be considered in ALL Ph+ treatment, because they appear to be more effective in the suppression of BCR-ABL1 kinase activity [20,21,22]. In addition, case reports have suggested that acquired T315l mutations can define resistance to dasatinib [23]. A few reports also failed to demonstrate TKI as having a positive impact on overall survival [24]. Nevertheless, the majority of patients in these studies were adults, which probably influenced the results [25,26]. Most of the reports on ALL Ph+ therapies have originated from Europe, which is a possible limitation. However, the Ma et al. study on 67 Southeast Asian patients (aged 8–62 years) provided convincing evidence that the biological response to TKI treatment in ALL Ph+ is comparable with that found in Caucasian patients. In that study, a major factor that impacted clinical outcome was the *BCR–ABL1* fusion transcript. Among 67 patients, 63% (*n* = 42) harbored the p190 transcript and 18% (*n* = 12) the p210 transcript. In 13% (*n* = 9) of the patients both p210 and 190 showed changes, which is very rare in studies on Caucasian patients [27]. The Japanese Pediatric Leukemia/Lymphoma Study Group (JPLSG) investigated the role of imatinib in ALL Ph+ pediatric patients (median age 7 years old) immediately before hematopoietic stem cell transplantation (HSCT). The treatment protocol consisted of the induction phase, consolidation phase, reinduction phase, two weeks of imatinib monotherapy, and the HSCT phase. The 4-year OS rate among all patients was 78.1 ± 6.5% [28].

## 3. Future Directions in Treatment

Before the era of tyrosine kinase inhibitors, the treatment of ALL Ph+ patients often failed, and outcomes were frequently poor. Imatinib allowed patients to achieve greater overall survival, and there is hope now for new strategies, such as second and third generation TKIs, bi-specific monoclonal antibodies, retinoids, and *BCL-2* inhibitors. Dasatinib and nilotinib are second-generation tyrosine kinase inhibitors that were developed to overcome resistance to imatinib. Dasatinib is 300 times more potent than imatinib at blocking ABL kinase activity; it accumulates in the central nervous system and can successfully be used in most patients with imatinib resistance. The Children’s Oncology Group trial tested the safety and feasibility of adding dasatinib to intensive chemotherapy from Day 15 of induction in patients with ALL Ph+, aged 1 to 30 years. The five-year overall survival in the dasatinib group was 86% ± 5% and 87% ± 5% for standard-risk patients [21]. The first randomized clinical study, comparing the efficacy of imatinib and dasatinib in children with ALL Ph+, was conducted by the Chinese Children’s Cancer Group (CCCG-ALL-2015). After starting dexamethasone, the ALL Ph+ patients began to receive dasatinib (80 mg/m^2^ per day) or imatinib mesylate (300 mg/m^2^ per day), and this continued until the end of therapy. The 4-year overall survival rate was 69.2% in the imatinib group vs. 88.4% in the dasatinib group [29]. Lastly, nilotinib is a highly specific TKI that can be considered as a therapeutic option for imatinib-resistant ALL Ph+ patients [30]. Kantarjian et al. suggested that nilotinib has limited efficiency in this subgroup, because only 2 of 13 patients in their study had a response to the drug [31]. However, there is no available treatment for patients with primary or secondary resistance to dasatinib or nilotinib, and a third generation TKI, ponatinib, could be effective in these cases. To our knowledge, there are no studies on ponatinib treatment in childhood Ph+ ALL, but in adults, ponatinib has shown significant clinical activity in a limited sample (*n* = 5). Therefore, more research is needed to more comprehensively evaluate the therapeutic value of ponatinib [32,33].

Blinatumomab is an anti-CD3 and anti-CD19 bispecific monoclonal antibody. Blinatumomab activates T cells with its anti-CD3 group and binds to tumor cells with its anti-CD19 group, to promote cellular cytotoxicity. Foà et al. conducted a Phase 2 single-group trial of first-line therapy in adults with newly diagnosed ALL Ph+. After induction with dasatinib plus glucocorticoids, two cycles of blinatumomab were administered. The results showed that at the end of dasatinib induction therapy, 29% of the patients had a molecular response; however, this percentage increased to 60% after two cycles of blinatumomab and increased even more after additional blinatumomab cycles. The OS after 18 months was 95% and the DFS was 88%; however, the DFS was lower among patients with *IKZF1* or *IKZF1^plus^* alterations [34].

Focal adhesion kinase (FAK) is a non-receptor-type tyrosine kinase that is constitutively activated in ALL Ph+. Hu et al. suggested that FAK is critical for leukemogenesis, and FAK inhibitors might be interesting candidates for B cell acute lymphoblastic leukemia treatment [35]. In an experimental study on mouse models of *BCR-ABL1 positive* leukemia, detection of *IKZF1* promoted the development of an aggressive lymphoid leukemia. Churchman et al. revealed that *IKZF1* alterations are the result of the gain of stem cell-like features, such as self-renewal and increased bone marrow stromal adhesion. Retinoid receptor agonists reversed this phenotype by inducing the expression of *IKZF1*. This stimulated the abrogation of adhesion and self-renewal, stopped the cell cycle, and reduced proliferation, without direct cytotoxicity. Using retinoids potentiated the activity of dasatinib in mouse *BCR-ABL1* ALL, representing a promising therapeutic option in *IKZF1*-mutated ALL [36]. The B-cell lymphoma 2 (BCL-2) protein family plays a significant role in the intrinsic mitochondrial apoptosis pathway, through interactions between pro- and antiapoptotic proteins. Direct targeting of *BCL*-2 constitutes a promising approach for treating leukemia. Venetoclax is an oral, highly selective BCL-2 inhibitor that has shown activity in BCL-2–dependent hematologic malignancies (*BCR/ABL1*-positive ALL, d T-ALL, *KMT2A-*rearranged ALL, hypodiploid) ALL. Preclinical study results suggested that ALL cells were dependent on both BCL-2 and BCL-XL. Adults with relapsed/refractory ALL or lymphoblastic lymphoma were treated with venetoclax and navitoclax (BCL-2 and BCL-XL inhibitor). Among 47 heavily pre-treated patients, the complete remission rate was 60%, which confirms that it could be an effective treatment option in the future [37,38,39].

## 4. *IKZF1* Deletions

Over the last few years, the treatment of childhood ALL has significantly improved, but precise awareness of the risk factors remains key in order to estimate the prognosis [2]. Recent discoveries have helped us to increase our knowledge about ALL based on genetics. Nowadays, treatment of each patient begins with genetic diagnostics of the genomic alterations using karyotyping, fluorescent in situ hybridization (FISH), multiplex ligation probe-dependent amplification (MLPA), microarrays, or next generating sequencing (NGS) [39]. Gene expression profiling studies have indicated that ALL Ph+ patients frequently carry co-occurring mutations (e.g., *IKZF1*, *PAX5*, *EBF1*) with *BCR-ABL1* fusion. Much effort in recent years has focused on screening for the gene that defines ALL patient’s outcomes. According to genetic changes, most subtypes of ALL have been identified to have mutations in the genes encoding epigenetic regulators and chromatin-modifying proteins. Several mutations acquired at relapse were detected in subclones at diagnosis, suggesting that the mutations may be responsible for resistance to therapy. The mutation of epigenetic modifiers, such as CREBBP, is detected in 18.3% of relapse ALL cases [40]. However, the most common finding is *IKZF1* gene alterations. The report of Mullighan et al. added to the existing knowledge that *IKZF1* mutations are often (87.5%) accompanied by *BCR-ABL1* rearrangements [41]. According to the age range, *IKZF1* alterations in pediatric ALL Ph+ occur in up to 50% of cases [41,42], and this increases to 84% among adult ALL Ph+ [43]. The *IKZF1* gene (OMIM*603023) is located on the short arm of Chromosome 7 (region p12.2) and consists of eight exons. The *IKZF1* (Ikaros Family Zinc Finger 1) gene encodes the transcription factor Ikaros, which is associated with chromatin remodeling and regulation of lymphocyte differentiation [44]. Ikaros has six zinc fingers, four of which are located in the DNA-binding domain encoded by Exons 4 to 6, and they are necessary to sustain Ikaros tumor-suppressor function. The rest of the zinc fingers are determined by Exon 8 and mediate the dimerization of Ikaros either as a homodimer or with other transcription factors of the Ikaros Zinc Finger family group [45]. Loss of Ikaros activity decreases sensitivity to tyrosine kinase inhibitors and deregulates the cascade of lymphoid genes in the hematopoietic stem cells, which normally stimulate differentiation into all three of the major hemolymphoid lineages [46]. There are three functional types of *IKZF1* mutations. The most common alterations (55%) are haploinsufficiency, when one copy of the *IKZF1* gene is inactivated or deleted, and the remaining allele of the gene is not adequate to produce the gene product, which is needed to preserve normal function. The second (33%) type is a dominant-negative form of Ikaros, made by an in-frame deletion of Exons 4 to 7 [47]. *IKZF1* deletion is associated with higher levels of white blood cells, older age at diagnosis, and MRD [48]. The many defined genetic alternations and markers related to *IKZF1* deletion have led to the need for a new subgroup, termed *IKZF1^plus^*. *IKZF1*^plus^ emphasizes the importance of moving from prognostic factors to a prognostic profile. *IKZF1*^plus^ is defined as an *IKZF1* deletion co-occurring with deletions in *CDKN2A* or *CDKN2B* (only homozygous deletions) or the *PAX5* or PAR1 region *(P2RY8-CRLF2*), in the absence of *ERG* deletion. *IKZF1*^plus^ patients have a 5-year EFS of 53 ± 6%, compared with 79 ± 5% in patients with *IKZF1* deletion or 87 ± 1% in patients who lack any *IKZF1* deletion. *IKZF1*^plus^ is a strong prognostic factor, but only in patients who are still carrying measurable MRD of a leukemic cell load exceeding 10^−4^ after induction treatment [49]. This relationship has already been underlined as a high-risk stratification criterion in the current frontline AIEOP-BFM ALL 2017 protocol for ALL treatment [50]. Moreover, research on the characteristics that unify *IKZF1*^plus^ patients as a subgroup has found the *GATA3* single-nucleotide variant rs382466245 to be enriched within *IKZF1*^plus^ patients. This may be the background to future studies into *IKZF1*^plus^ inheritance [51]. Inherited *GATA3* variants are associated with Ph-like childhood acute lymphoblastic leukemia, and risk of relapse [52]. The Malaysia–Singapore ALL 2010 (MS2010) study was one of the first trials focusing on effective chemotherapy in ALL Ph+ pediatric patients with *IKZF1* deletions. All patients with ALL Ph+ were treated with imatinib, starting from Day 15 of induction, for a total of 2 years. Likely because of imatinib administration, the 5-year cumulative incidence of relapse (CIR) decreased from 66.7% (*n* = 12) to 20% (*n* = 10) compared with MS2003, an earlier version of the study [53,54]. Interestingly, Palmi and colleagues put forward the hypothesis that the poor outcomes of the patients with *IKZF1* deletions is evidence of genomic instability in aggressive leukemic clones, rather than a result of reduced *IKZF1* function caused by *IKZF1* haploinsufficiency, as earlier trials suggested [55]. Studies on monozygotic twins have suggested that ALL Ph+ can be an effect of prenatal *BCR-ABL1* fusion. Additionally, in a pair where both children had *BCR/ABL1* fusion, one twin with an *IKZF1* mutation was diagnosed with ALL and died later, and the other remained healthy. Based on the research results, we assume that *IKZF1* mutations may be associated with a poor prognosis. Moreover, this data suggests that *BCR-ABL1* gene fusion can already exist prenatally [56]. The Children’s Oncology study confirmed that *IKZF1* alteration is strongly associated with poor outcomes in *BCR-ABL1*-negative patients as well, which indicates that *IKZF1* determines leukemogenesis and response to therapy [57,58,59]. Unfortunately, because of ALL Ph+ heterogeneity, *IKZF1* mutation is not the only important aspect of outcome prediction. Research in various centers around the world may indicate genetic predisposition of various ethnic groups. A study from India on 132 patients (aged from 2 months to 67 years, median age 8.5 years) confirmed that *IKZF1* is a commonly detected mutation at 26.5% (*n* = 43), and is associated with older age and higher induction failure rates [60]. Similar results were observed in a study from China, where patients (aged 14–70) with *IKZF1* deletions had a higher relapse risk and worse survival rate. These data also suggested that transplants could overcome the adverse impact of *IKZF1* deletions, although these results need to be confirmed in a randomized study in the future [61].

## 5. Molecular Background of Ph-Like

High-throughput genomic studies have enabled the identification of another genetic alteration, providing the rationale to test targeted therapies in genetically defined patient subsets. ALL Ph+ is defined by the t(9;22)(q34;q11) translocation that produces *BCR-ABL1*, a constitutively active tyrosine kinase. Based on gene expression profiling studies, a new subtype of ALL was described, named Philadelphia chromosome-like ALL [62]. Ph-like ALL is characterized by a gene expression profile that is extremely similar to ALL Ph+, but lacking the exact *BCR-ABL1* fusion. Therefore, among ALL subtypes, Ph-like ALL is the most heterogeneous, which poses challenges in diagnosis and treatment. Copy number changes, chromosomal rearrangements, aneuploidy, and deregulated gene expression are only some types of genetic alterations. Male and Down Syndrome patients more often suffer from Ph-like ALL [63]. Ph-like ALL incidence increases with age and is indicated in 15% of B-ALL pediatric cases, rising to 21% in adolescents and up to 27% in young adults with B-ALL [64]. However, after young adulthood, the frequency of Ph-like ALL decreases [65]. This is the opposite to the frequency of Ph-positive ALL, which is known to continually increase with age [66]. Studies have confirmed that more than 80% of Ph-like cases are associated with the kinase activating pathway or cytokine receptor alteration [67,68]. The heterogeneity of genetic variants is enormous; however, the two main groups are based on activation of the ABL-class (*ABL1, ABL2, PDGFRA, PDGFRB*) or JAK-STAT (*IL7R*, *JAK1*, *JAK3*) alteration. Among Ph-like ALL patients, 10–14% have rearrangements of the ABL-class genes (*ABL1*, *ABL2*, *PDGFRA*, *PDGFRB*, *LYN*, *CSF1R*) other than *BCR-ABL1* [63,69]. Knowledge of ABL-class fusions helps to better determine the treatment of these patients. Patients with the *EBF1-PDGFRB* fusion have a significantly higher rate of MRD and induction failure [70]. In addition, the *EBF1* and *PAX5* genes are frequently deleted, but their prognostic significance is not fully understood. Results of the Therapeutically Applicable Research to Generate Effective Treatments (TARGET) study undertaken by the National Cancer Institute identified new gene mutations, including *CRLF2, IL-7R, JAK1,* and *JAK2,* which assign ALL patients to a high-risk group [71]. Another similarity to ALL Ph+ is a high frequency of *IKZF1* mutations, which is observed in 70–80% of Ph-like patients [72]. Additionally, half of Ph-like ALL patients have cytokine receptor-like factor 2 gene-*CRLF2* over-expression, and it is more often indicated in older children [63,68,73]. Furthermore, up to 50% of *CRLF2* cases harbor concomitant *JAK* mutations, the most popular of which is the R683G point mutation located in the pseudokinase domain of *JAK2* [74,75]. In contrast, some studies have suggested that in Ph-like patients, *ETV6-RUNX1* is more frequent in younger children compared with older children. Moreover, *ETV6-RUNX1* is also associated with a favorable outcome [76]. Furthermore, in 4% of Ph-like patients, *EPOR* rearrangements are indicated, which normally stimulate red blood cells development [77,78]. In the most recent Children’s Oncology Group (COG) study, Ph-like ALL patients’ 5-year event-free survival rate was 63%, compared with 86% in non-Ph-like cases [71]. An observation made by Weston et al. indicated that *BCR-ABL1*-like ALL patients with *EBF1-PDGFRB* rearrangements (identified in 8% of patients) could be successfully treated with TKI in cases refractory to conventional four-drug induction therapy [79,80]. Various findings exist as to the occurrence of *IKZF1* mutations in the BCR-ABL1 gene in ALL Ph-like patients. The St. Jude Children’s Research Hospital and Children’s Oncology Group found them in 80% to 90% of cases, but Van Der Veer identified *IKZF1* mutations in 40% of patients [81,82]. Nevertheless, *IKZF1* mutations may predict an unfavorable outcome in ALL Ph+, as well as in Ph-like ALL. The results of a study conducted in Taiwan suggested that the incidence of Ph-like ALL, as well as *IKZF1* deletion in B-cell ALL patients, are lower in Taiwan than in Europe. More attention should be paid to the diversity of frequency of genetic alternations in different ethnicity groups [83]. It has long been known that some ploidy genomes or number of chromosomes impact on ALL outcome. We can classify Ph-like patients based on the number of chromosomes. Hyperdiploidy is characteristic of Chromosomes 51–65, high hypodiploidy of 40–44, low hypodiploidy of 30–39, and near-haploidy is typical for chromosomes <30. Hypodiploidy is rare and occurs in around 6% of ALL patients, of which the majority (80%) have 45 chromosomes [84,85]. High hyperdiploidy is associated with a better outcome; however, low hypodiploidy and near-haploidy are both linked with high rates of relapse [14,86,87]. Hypodiploidy, as well as near haploid ALL, demonstrate activation of the Ras and PI3K signaling pathways, and are sensitive to PI3K inhibitors, which may be the ultimate aim of targeted therapy.

The 2016 World Health Organization (WHO) classification of hematopoietic and lymphoid tissues highlights progress in the identification of unique biomarkers and gene expression, which has improved the diagnostic criteria [88]. In GMALL (the German Multicenter Study Group for Adult ALL), *IGH-CRLF2* rearrangements and mutations in *JAK2* were found exclusively in the Ph-like ALL subgroup. The authors suggested that screening gene expression analysis of these genes could be an easy way to identify Ph-like ALL patients. Ph-like ALL is a challenge to diagnose [89]. Boer et al. analyzed the gene expression of patients with newly diagnosed ALL and identified *BCR-ABL1*-like in 15% of precursor B-ALL cases. However, an analysis of the karyotype of *BCR-ABL1*-like patients did not reveal a common genetic feature. Patients did not present anomalies such as hyperdiploidy, *ETV6–RUNX1* translocation, *TCF3-*rearrangement, *KMT2A*-rearrangement, or *BCR–ABL1* translocation. However, array-CGH (comparative genomic hybridization) tests revealed the most common deletions in Chromosomes 9p and 20q, and local amplification of regions of Chromosomes 21q21–q22 [62].

## 6. Treatment of Ph-Like

The results of allogeneic stem cell transplants in Ph-like ALL in children, as well as adults, show it is often unsuccessful, and new therapies could be life changing [90]. Moreover, Boer et al. suggested that *BCR-ABL1*-like leukemic cells have special properties that make them 1.6 times more resistant to daunorubicin and 73 times more resistant to L-asparaginase than other precursor B-ALL cases. Interestingly, there were no differences in adverse cytotoxic effects of vincristine or prednisolone [62]. The role of TKI with intensive chemotherapy treatment in Ph-like ALL patients has not been established yet, but promising results in ALL Ph+ patients lead us to expect therapeutic success. Close to 20% of B-ALL cases showed gene rearrangements of *CRL2*, *ABL1*, *JAK2*, *PDGFRB*, *JAK1*, and *JAK2*, which activate kinase signaling [91]. The kinase fusion and partner genes identified in Ph-like ALL are presented in Table 2. Due to the high occurrence of kinase-activating mutations in Ph-like, there is a possibility that unique approaches in precision medicine could drive tailored treatment [82]. Data from various groups have clearly shown that the *ABL1* alteration can be treated by TKI, and JAK-STAT mutations by *JAK* inhibitors [92]. Tanasi et al. found that patients with ABL-class kinase fusion treated with TKI frontline or at relapse demonstrated a better MRD response and overall survival. Moreover, the authors reported significantly improved outcomes among children and adolescents in comparison to adults [93]. The results of a multicenter worldwide retrospective study conducted by the Ponte di Lego group strengthens the evidence regarding the use of TKIs. A total of 122 patients with newly diagnosed *ABL*-class fusion B-cell ALL underwent treatment that did not contain tyrosine-kinase inhibitors. In this group, the 5-year event-free survival was 59.1%, and the 5-year overall survival was 76.1% [94]. By contrast, in Cario’s study, patients with *ABL*-class fusion were divided into two groups. The first group (*n* = 33) was treated according to AIEOP-BFM (Associazione Italiana di Ematologia-Oncologia Pediatrica–Berlin-Frankfurt-Münster) protocols, without TKI, and the second group (*n* = 13) received TKI (imatinib in eight and dasatinib in five cases) during different phases of treatment. Interestingly, their 5-year EFS and 5-year OS did not differ significantly compared with the no-TKI group [9]. However, the non-randomized UKALL2011 trial demonstrated a reduced risk of relapse for *ABL*-class fusion patients with MRD ≥ 1% treated with adjuvant TKI, without a significantly increased risk of severe toxicity [95]. Due to the diversity and small number of Ph-like patients, it was hard to plan a randomized clinical trial on the basis of developing treatment standards. There is a need for international, multicenter collaboration to achieve this [23]. In this context, successful treatment in Ph-like patients remains challenging. However, early indication of molecular treatment targets and future studies on TKI could lead to progress in the near future in developing treatment protocols.

## 7. Conclusions

The mechanisms of the detailed molecular changes in ALL Ph+, especially the sequence of changes, as well as the genetic risk factors, have not yet been established. It is expected that, in the next few years, the genomic landscape of ALL Ph+ will be completely described, which will provide the basis for further research into treatments. Molecular research allowed us to distinguish a new group of ALL, Ph-like ALL, which is characterized by broad-spectrum genetic alterations, but does not have an exact *BCRL/ABL1* fusion. Identification of genetic mutations can help predict the prognosis, but classification to the appropriate risk group still remains a challenge. IKZF1 deletion is a predictor of adverse outcomes in pediatric ALLs. ALL Ph+ is characterized by poor outcomes, but more recently, tyrosine kinase inhibitors appear to have improved patient outcomes. The spectrum of drugs that hold promise as therapeutic options for treating ALL Ph+ is expanding. Although ALL Ph+ treatment is standardized by the EsPhALL protocol, we are still waiting for treatment recommendations for Ph-like ALL. Ph-like heterogeneity is a limiting factor in the conduction of randomized controlled trials, which are nevertheless required to establish treatment approaches.

## Figures and Tables

**Table 1:** Comparison of treatment results without the use of TKIs, and the EsPhALL 2004 and 2010 protocols.

Treatment Protocols		DFS ^1^ (%)	EFS ^2^ (%)	OS ^3^ (%)	Age < 10 Years (%)	Age ≥ 10 Years (%)	Serious Adverse Events (%)	p190 Transcript (%)	p210 Transcript (%)	MRD ^4^ (%)
Without using TKIs study 1986–1996 (*n* = 326)	-	-	28.3	40.3	64	36	-	-	-	-
EsPhALL2004	Good risk imatinib group (*n* = 46)	72.9	-	78.5	61	39	28	92	8	63
	Good risk no imatinib group (*n* = 44)	61.7	-		64	46	32	90	10	35
	Poor risk group (*n* = 70)	53.5	-	62.9	41	59	34	78	23	96
EsPhALL2010	Good risk group (*n* = 102)	-	62.7	75.7	65	35	50	78	22	15
	Poor risk group (*n* = 53)	-	46.3	63.6	51	49	55	98	2	52

^1^ DFS, the 4-year disease free survival (%); ^2^ EFS, the 5-year event free survival; ^3^ OS, the 5-year overall survival; ^4^ MRD, minimal residual disease (at end of induction ≥ 5 × 10^−4^).

**Table 2:** Kinase fusion and partner genes identified in Ph-like ALL, and therapy strategies.

Kinase Fusion Identified in Ph-Like ALL	Fusion Partners Gene	Treatment	References
ABL1	*CENPC, ETV6, LSM141, NUP153, NUP214, RANBP2, RSCD1, ZMIZ1, FOXP1, LSM14A, NUP153, NUP214, RANBP2, RCSD1, SFPQ, SNX1, SNX, SPTAN1, ZC3HAV1*	Imatinib, Dasatinib, GNF2 GNF5	[82,96,97]
ABL2	*PAG1, RSCD1, ZC3HAV1,*	Imatinib/Dasatinib	[64]
PDGFRB	*SSBP2, TBL1XR1, EBF1, TNIP1, ZEB2, ATF71P, ETV6, PAX5, PCM1, PPF1BP1, RFX3, SSBP2, STRN3*	Dasatinib	[79,80,82]
PDGFRA	*FIP1L1*	Dasatinib	[98]
CSF1R	*SSBP2*	Dasatinib	[64]
CRLF2	*IGH, P2RY8*	JAK2 inhibitor	[74,99]
LYN	*GATAD2A, NCOR1*	Imatinib/Dasatinib	[98]
JAK2	*BCR, PAX5, PCM1, RFX3, USP25, ZNF274, ATF1, EBF1, ETV6, PAX5, PCM1, PPFIBP1, RFX3, SSBP2, STRN3, TERF2, TPR, USP25, ZNF274, GOLGA5, SMU1, SNX29, ZNF340*	JAK2 inhibitor	[82,100,101,102]
EPOR	*IGH, IGK, LAIR1, THADA*	JAK2 inhibitor	[64,82,98]
TYK2	*MYB, SMARCA4,*	TYK2 inhibitor	[64]
TSLP	*IQGAP2*	JAK2 inhibitor	[64]
DGKH	*ZFAND3*	Unknow	[64]
IL2RB	*MYH9*	JAK1/JAK3 inhibitor or both	[64]
NTRK3	*ETV6*	TRK inhibitor, Crizotinib	[103]
PTK2B	*KDM6A, STAG2, TMEM2*	FAK inhibitor	[64]
FLT3	*ZMYM2*	FLT3 inhibitor	[104]
FGFR1	*BCR*	Sorafenib, Dasatinib, Ponatinib	[105]
BLNK	*DNNT*	Unknown	[106]

## Data Availability

No new data were created or analyzed in this study. Data sharing is not applicable to this article.

## Abbreviations

ABL1	V-abl Abelson murine leukemia viral oncogene homolog 1
ABL2	V-abl Abelson murine leukemia viral oncogene homolog 2
AIEOP-BFM	Associazione Italiana di Ematologia-Oncologia Pediatrica–Berlin-Frankfurt-Münster
ALL	acute lymphoblastic leukemia
ALLO	hsct-allogenic hematopoietic stem cell transplantation
ATF1	activating transcription factor 1
ATF71P	activating transcription factor 7-interacting protein
B-ALL	B-cells Acute Lymphoblastic Leukemia
BCR	breakpoint cluster region
BCR/ABL1	BCR and ABL fusion gene
BLNK	B-cell linker protein
CENPC	centromeric protein c1
CIR	cumulative incidence of relapse
COG	children oncology group
CR1	first complete remission
CRLF2	cytokine receptor-like factor 2
CSF1R	Colony Stimulating Factor 1 Receptor
DFS	disease-free survival
DGKH	diacylglycerol kinase, ETA, 130-KD
EBF1	early B-cell factor 1
EFS	event-free survival
EPOR	erythropoietin receptor
ETV6	ets variant transcription factor 6
ETV6–RUNX1	*ETV6* and *RUNX1* fusion gene
FGFR1	fibroblast growth factor receptor 1
FIP1L1	actor interacting with PAPOLA and CPSF1
FISH	fluorescence in situ hybridization
FLT3	FMS-related tyrosine kinase 3
FOXP1	forkhead box P1
FRS	event-free survival
GATAD2A	gata zinc finger domain-containing protein A2
GNF2	allosteric Bcr-abl inhibitors
GNF5	allosteric inhibitor of Bcr-Abl
GOLGA5	golgin A5
HSCT	hematopoietic sterm cell transplantation
IGH	immunoglobulin heavy
IGK	immunoglobulin kappa locus
IL2RB	interleukin 2 receptor, beta
IL7R	interleukin 7 receptor alpha chain
IQGAP2	IQ motif-containing GTPase-activating protein 2
JAK1	Janus kinase 1
JAK2	Janus kinase 2
JAK3	Janus kinase 3
JAK-STAT	Janus kinase-signal transducer and activator of transcription
KDM6A	lysine demethylase 6A
LAIR1	leukocyte associated immunoglobulin like receptor 1
LSM14A	mRNA processing body assembly factor
LYN	LYN proto-oncogene
MRD	minimal residual disease
MS2010	Malaysia–Singapore ALL 2010 study
MYB	protooncogene, transcription factor
MYH9	myosin, heavy chain 9, nonmuscle
NCOR1	nuclear receptor corepressor 1
NGS	next generating sequencing
NTRK3	neurotrophic tyrosine kinase, receptor, type 3
NUP153	nucleoporin 153
NUP214	nucleoporin 214
P2RY8	pyrimidinergic receptor P2Y, G protein coupled 8
PAG1	phosphoprotein associated with glycosphingolipid-enriched microdomains 1
PAX5	paired box gene 5
PCM1	pericentriolar material 1
PDGFRA	Platelet-derived growth factor receptor alpha
PDGFRB	Platelet-derived growth factor receptor beta
Ph	Philadelphia chromosome
Ph+-	Philadelphia chromosome positive
PPFIBP1	protein-tyrosine phosphatase, receptor type, F polypeptide-interacting protein-binding protein 1
PTK	protein tyrosine kinase
PTK2B	protein-tyrosine kinase 2, beta
RANBP2	ran-binding protein 2
RCSD1	RCSD domain containing 1
RFX3	regulatory factor 3
SFPQ	splicing factor proline- and glutamine-rich
SMARCA4	SWI/SNF-related, matrix-associated, actin-dependent regulator of chromatin, subfamily A, member 4
SMU1	DNA replication regulator and spliceosomal factor
SNX1	sorting nexin 1
SNX29	sorting nexin 29
SPTAN1	spectrin alpha, non-erythrocytic 1
SSBP2	single-stranded DNA-binding protein 2
STAG2	stromal antigen 2
STRN3	striatin, calmodulin-binding protein 3
TARGET	Therapeutically Applicable Research to Generate Effective Treatments
TBL1XR1	transducin-beta-like 1 receptor 1
TERF2	telomeric repeat-binding factor 2
THADA	thada armadillo repeat-containing protein
TKI	tyrosine kinase inhibitors
TMEM2	transmembrane protein 2
TNFAIP3	interacting protein 1
TNIP1	TNFAIP3 interacting protein 1
TPR	translocated promoter region
TSLP	thymic stroma lymphoprotein
TYK2	tyrosine kinase 2
USA	United States of America
USP25	ubiquitin-specific protease 25
ZC3HAV1	zinc finger CCCH-type containing, antiviral 1
ZEB2	zinc finger E box-binding homeobox 2
ZFAND3	zinc finger AN1 domain-containing protein 3
ZMIZ1	zinc finger miz-domain containing 1
ZMYM2	zinc finger, MYM-type 2
ZNF274	zinc finger protein 274
